# Supplementary material for: Mechanism of early light signaling by the carboxy-terminal output module of Arabidopsis phytochrome B
Source: Nat Commun. 2017 Dec 4;8:1905. doi: 10.1038/s41467-017-02062-6 (PMC5712524; doi:10.1038/s41467-017-02062-6)
Supplement: Supplementary file 1 — Supplementary Information [file 41467_2017_2062_MOESM1_ESM.pdf]

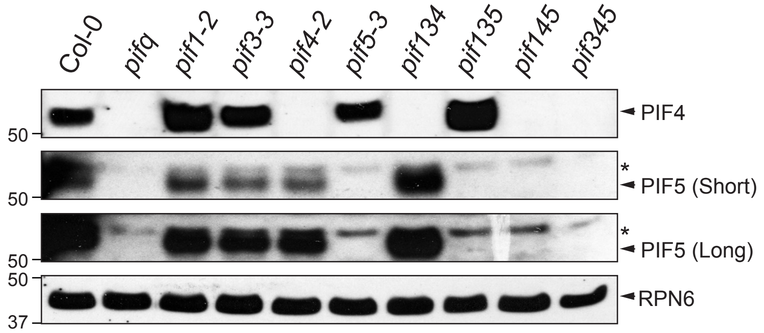


**Supplementary Figure 1. Test the specificity of two commercially available anti-PIF4 and -PIF5 antibodies by Agrisera.** Total protein samples from 4-d-old true-dark grown seedlings of Col-0, *pifq*, *pif1-2*, *pif3-3*, *pif4-2*, *pif5-3*, *pif134*, *pif135*, *pif145*, and *pif345* were subject to immunoblots using the anti-PIF4 or PIF5 antibodies. RPN6 was used as a loading control. Asterisks indicate non-specific bands. The anti-PIF4 antibody recognizes a band that is present in Col-0 and all *pif* mutants with the wild-type *PIF4* and is absent in all *pif4* knockout mutants, including *pifq*, *pif4-2*, *pif134*, *pif145*, and *pif345*. The anti-PIF5 antibody recognizes a band that is that is present in Col-0 and all *pif* mutants with the wild-type *PIF5* and is absent in all *pif5* knockout mutants, including *pifq*, *pif5-3*, *pif135*, *pif145*, and *pif345*. The short-exposure image shows that the PIF5 band is below a minor non-specific band; the long-exposure image shows that the non-specific band is present in pifq. Together, these results show that the anti-PIF4 and -PIF5 are specific for detecting PIF4 and PIF5, respectively.

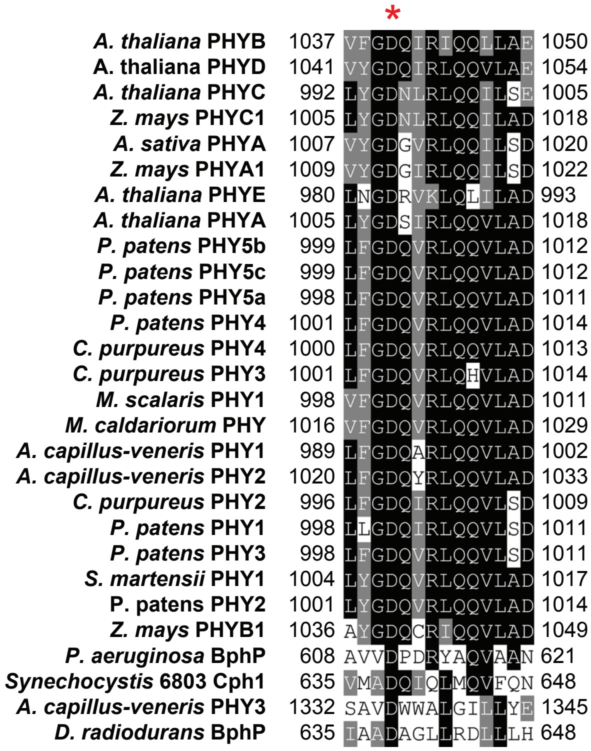


**Supplementary Figure 2. Sequence alignment of the amino acid sequences from plant, algal, and bacterial phytochromes flanking Asp 1040 of *Arabidopsis* PHYB.** The red asterisk indicates the conserved Asp. The sequence alignment was performed using ClustalW. Identical and similar amino acids are in black and grey background, respectively. The GenBank accession numbers for the selected sequences are: *A. thaliana* PHYA, AAA21351.1; *A. thaliana* PHYB, P14713.1; *A. thaliana* PHYC, CAA35223.1; *A. thaliana* PHYD, CAA54072.1; *A. thaliana* PHYE, CAA54075.1; *A. sativa* PHYA, AAA76820.1; *Z. mays* PHYA1, AAP06787.1; *Z. mays* PHYB1, AAP06788.1; *Z. mays* PHYC1, AAP06790.1; *A. capillus-veneris* PHY1, BAA31856.1; *A. capillus-veneris* PHY2, BAA33775.1; *A. capillus-veneris* PHY3, BAA36192.2; *S. martensii* PHY1, CAA43698.1; *C. purpureus* PHY2, AAB67863.1; *C. purpureus* PHY3, AAM94956.1; *C. purpureus* PHY4, ABW91071.1; *M. scalaris* PHY1, BAE20167.1; *P. patens* PHY1, EDQ57045.1; *P. patens* PHY2, EDQ52859.1; *P. patens* PHY3, EDQ69094.1; *P. patens* PHY4, EDQ61588.1; *P. patens* PHY5a, EDQ73867.1; *P. patens* PHY5b, EDQ67905.1; *P. patens* PHY5c, EDQ80816.1; *M. caldariorum* PHY, AAC49128.1; *Synechocystis* sp. PCC 6803 Cph1, Q55168.1; *P. aeruginosa* BphP, CTQ35777.1; *D. radiodurans* BphP, Q9RZA4.1.

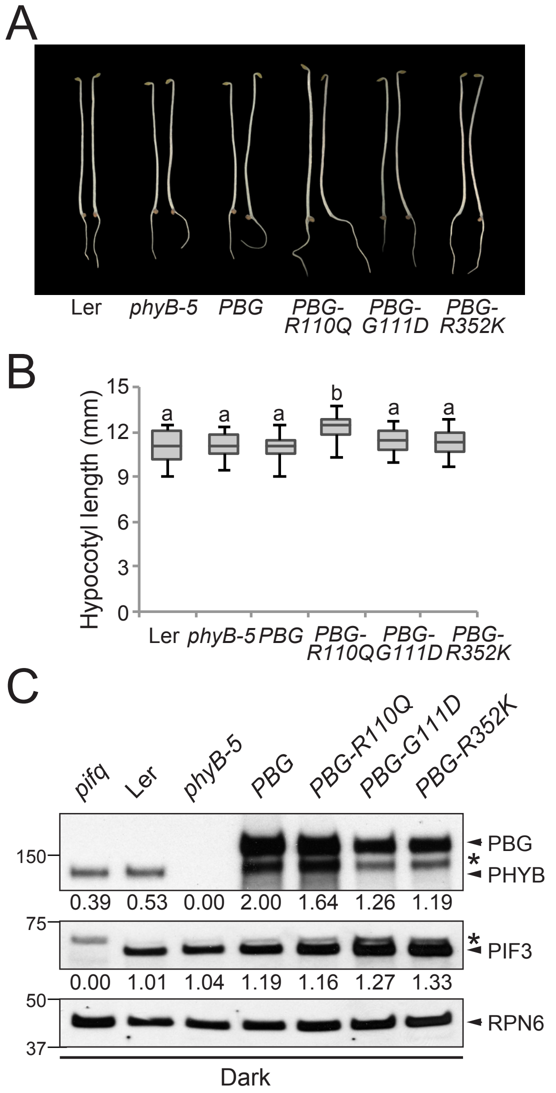


**Supplementary Figure 3. Phenotypes of *phyB-R110D*, *-R110D*, and *-R352K* mutants in the dark. (a)** Images of 4-d-old Ler, *phyB-5*, *PBG*, *PBG-R110Q*, *PBG-G111D*, and *PBG-R352K* lines grown in the dark. **(b)** Box and whisker plots of hypocotyl length measurements of the respective seedlings shown in (a). The boxes represent from 25th to 75th percentile; the bars equal to the median values. Samples with different letters show statistically significant differences in hypocotyl length (ANOVA, Tukey's HSD, $P < 0.05$, $n > 40$). **(c)** Immunoblots showing the levels of PHYB and PIF3 in 4-d-old Ler, *phyB-5*, *PBG*, *PBG-R110Q*, *PBG-G111D*, and *PBG-R352K* lines grown in darkness. RPN6 was used as a loading control. The relative levels of PHYB and PIF3 were normalized against the corresponding levels of RPN6 and are shown below the blots. Asterisks indicate nonspecific bands.

## Figure 1d

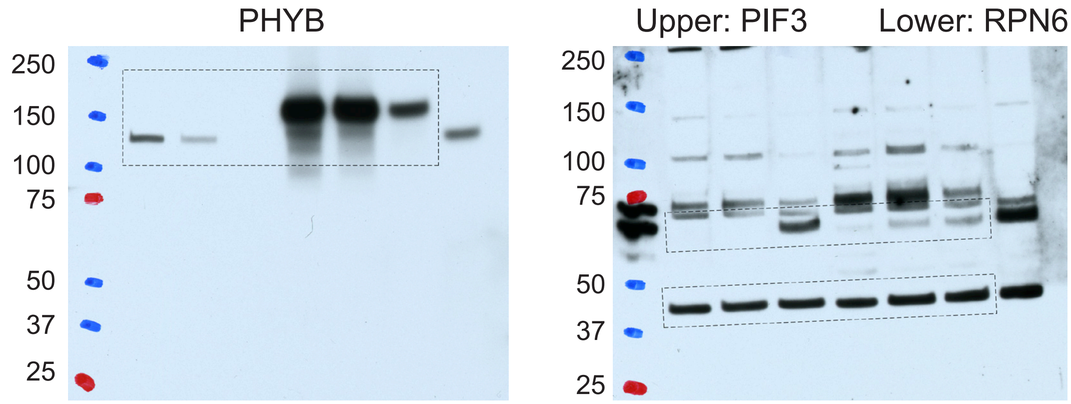


## Figure 1f

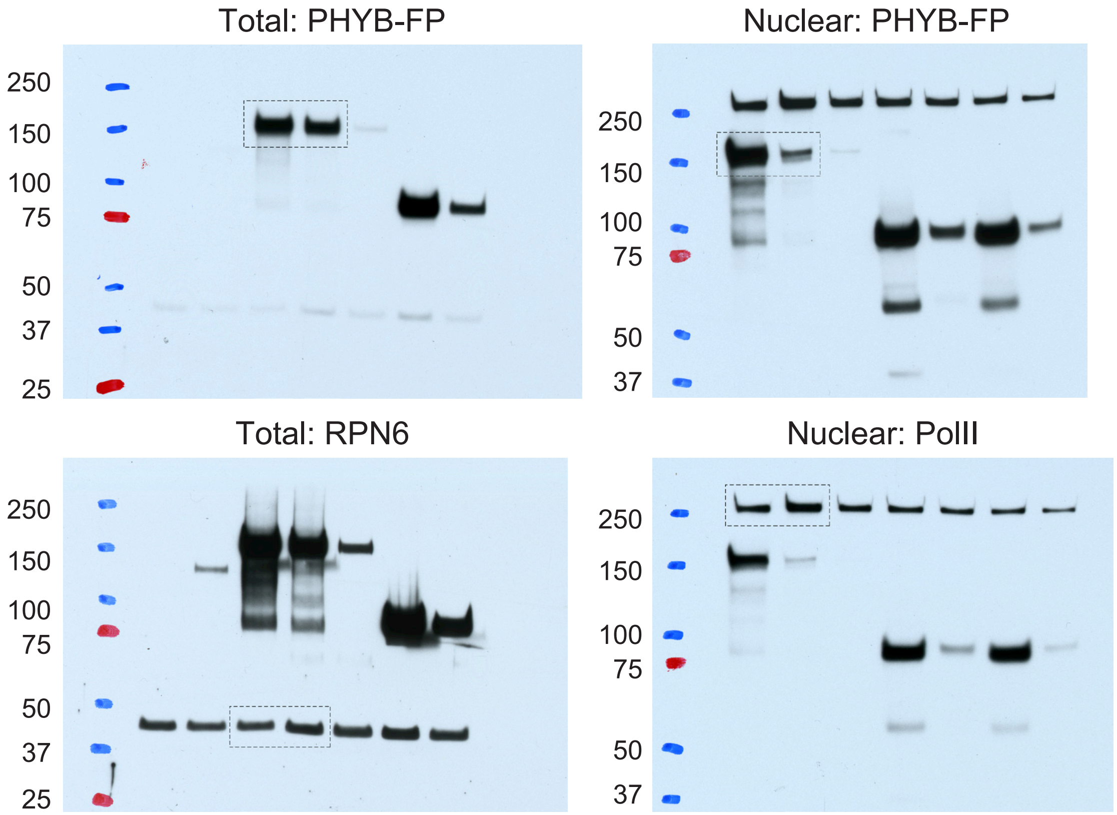


**Supplementary Figure 4. Scanned original images of immunoblots and gels.**

## Figure 2d

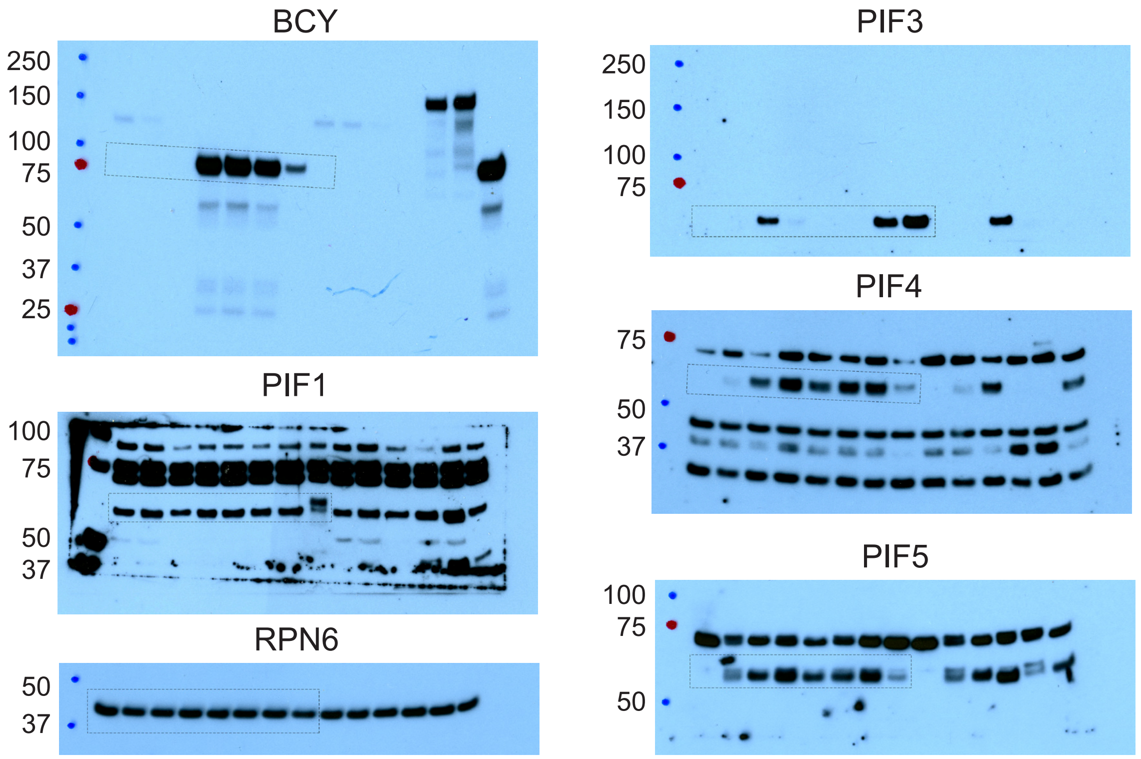


## Figure 2g

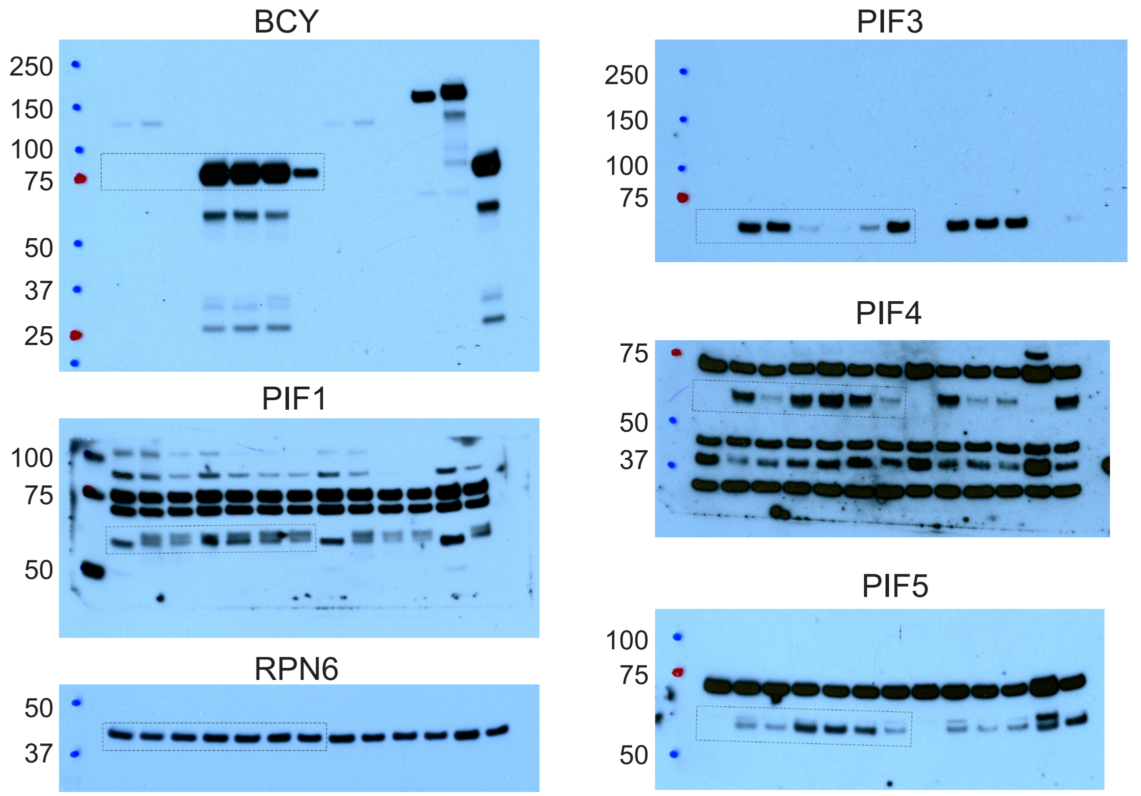


**Supplementary Figure 4. (continued)**

## Figure 3c

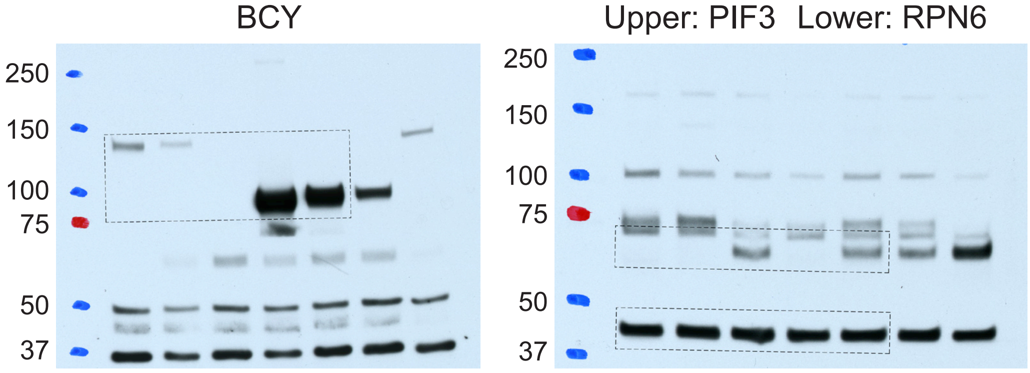


## Figure 3d

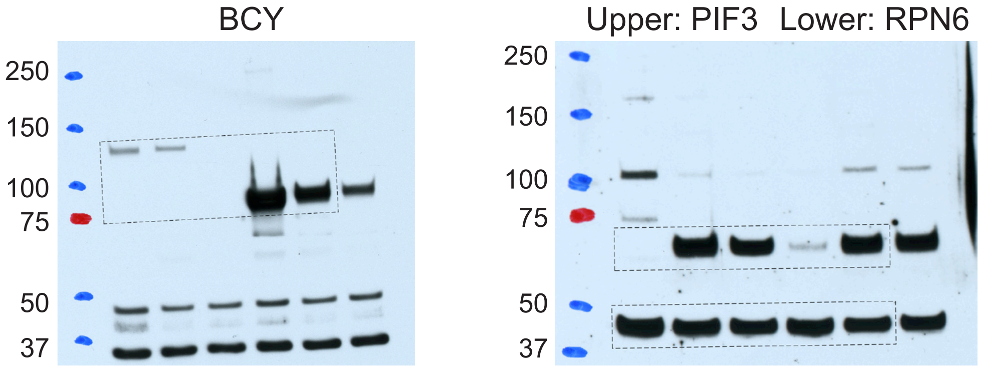


## Figure 3f

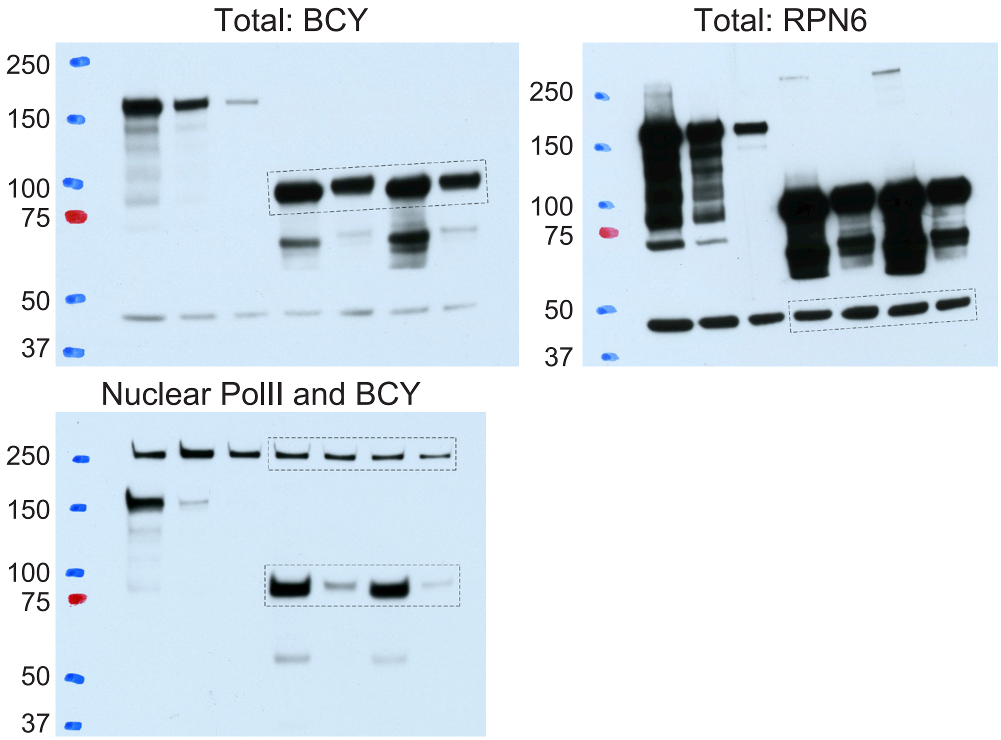


**Supplementary Figure 4. (continued)**

## Figure 4e

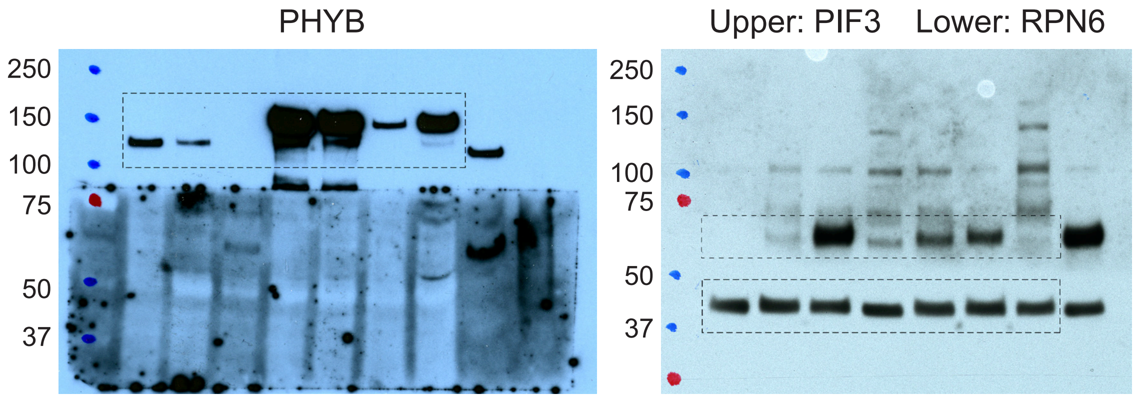


## Figure 4f (left panel)

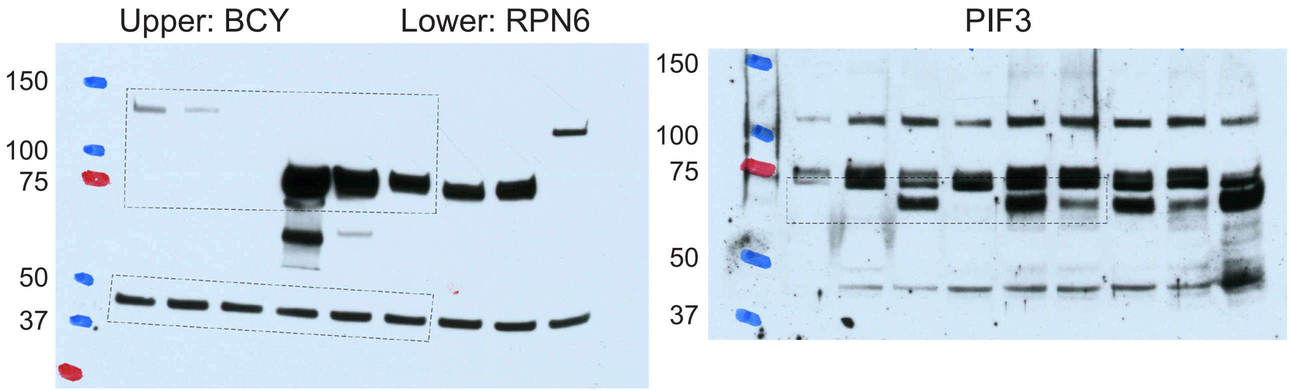


## Figure 4f (right panel)

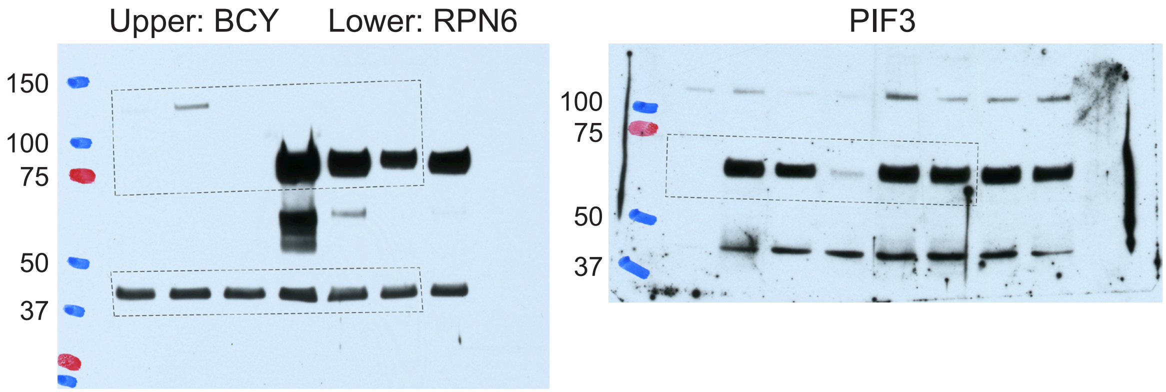


**Supplementary Figure 4. (continued)**

## Figure 7b

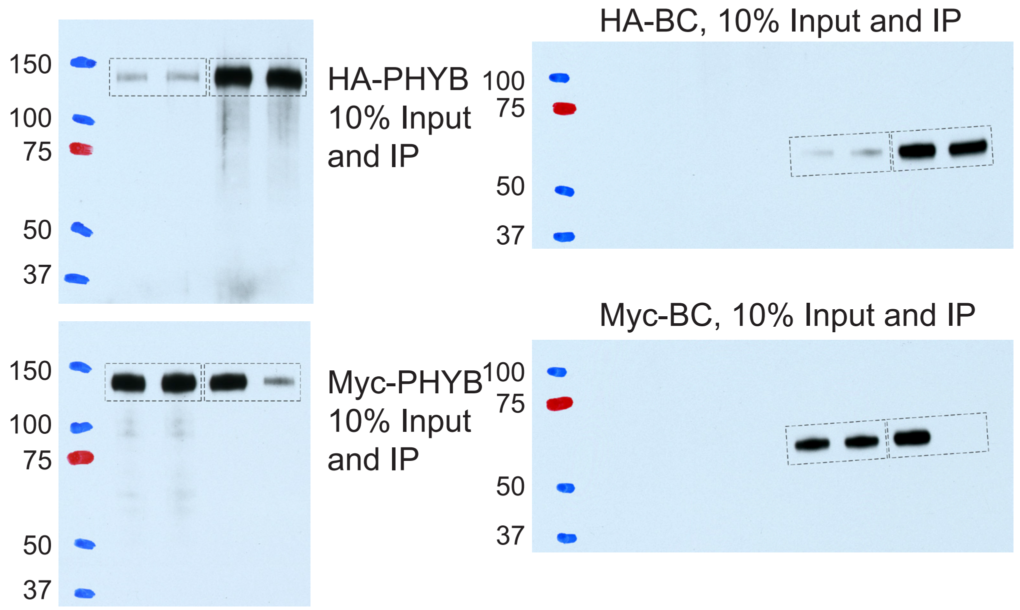


## Figure 7c

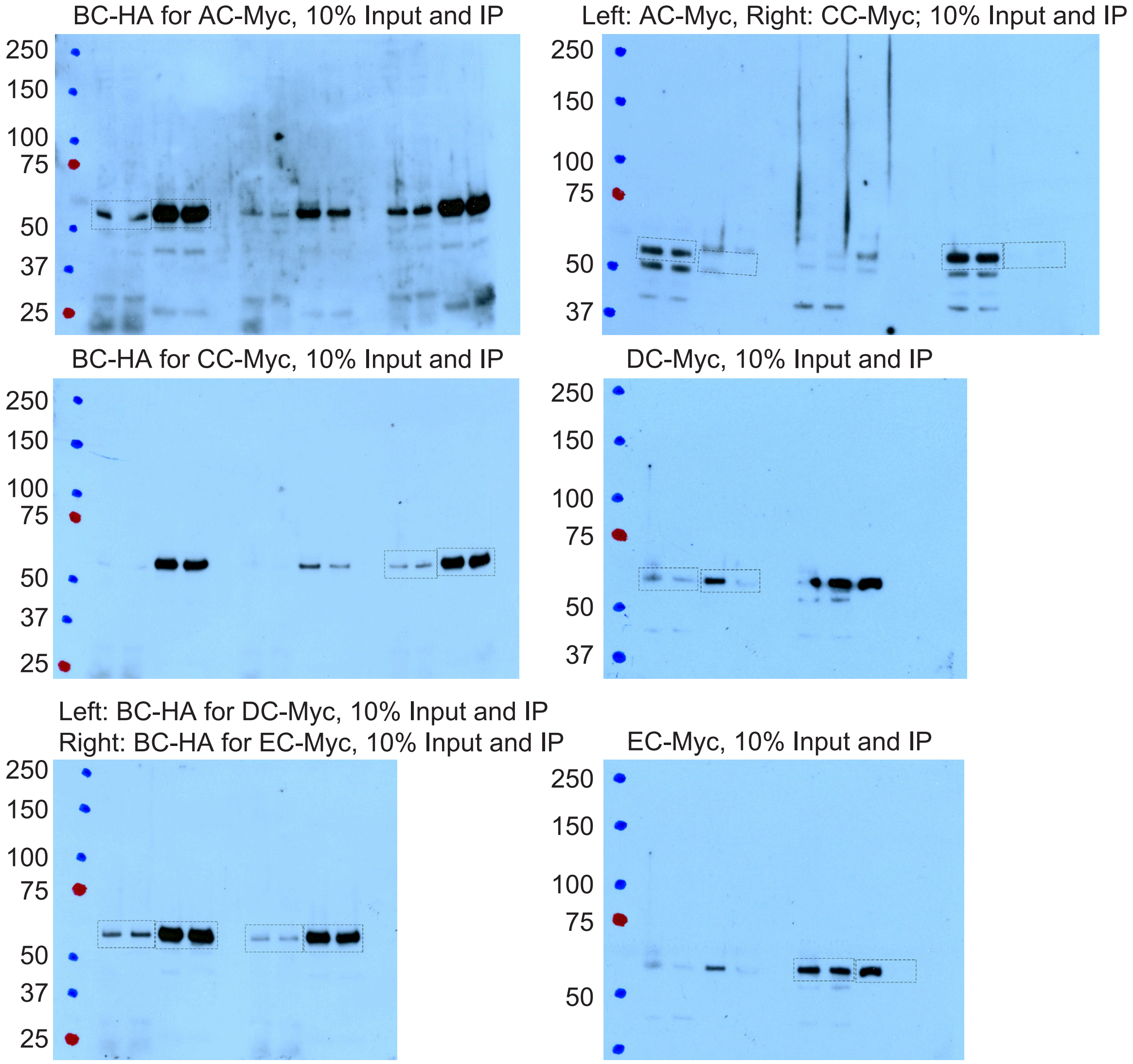


**Supplementary Figure 4. (continued)**

## Figure 8a

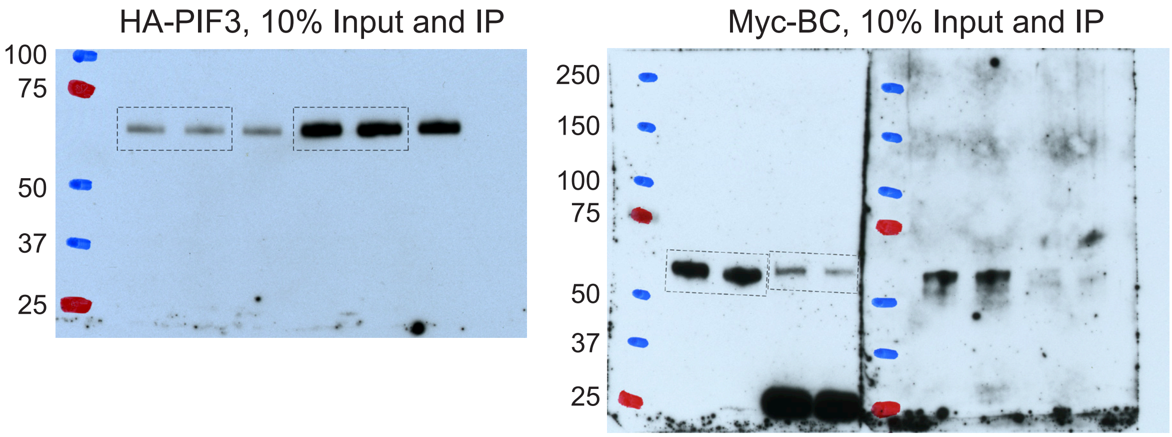


## Figure 8b

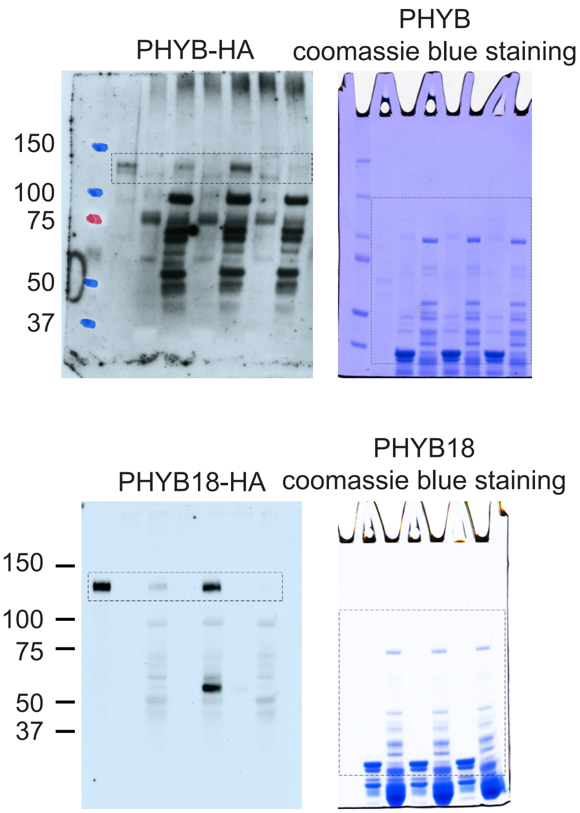


## Figure 8e

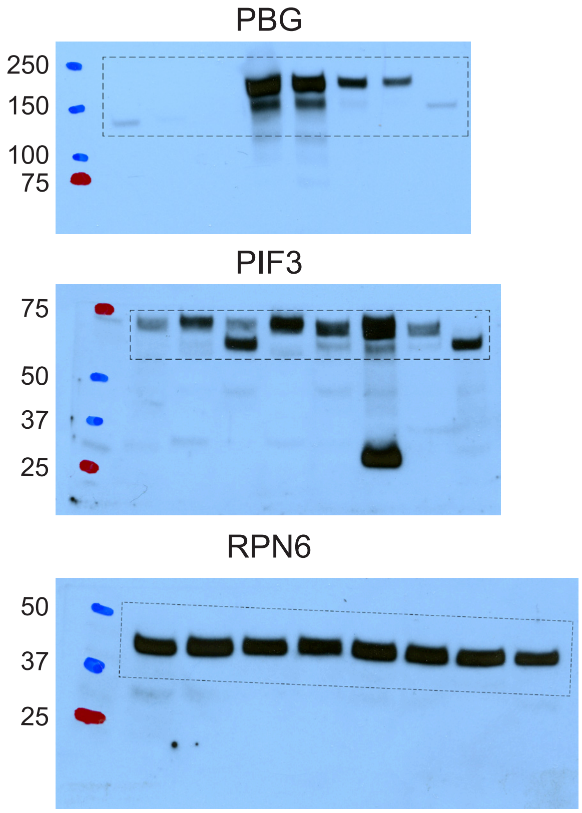


**Supplementary Figure 4. (continued)**

Supplementary Figure 3c

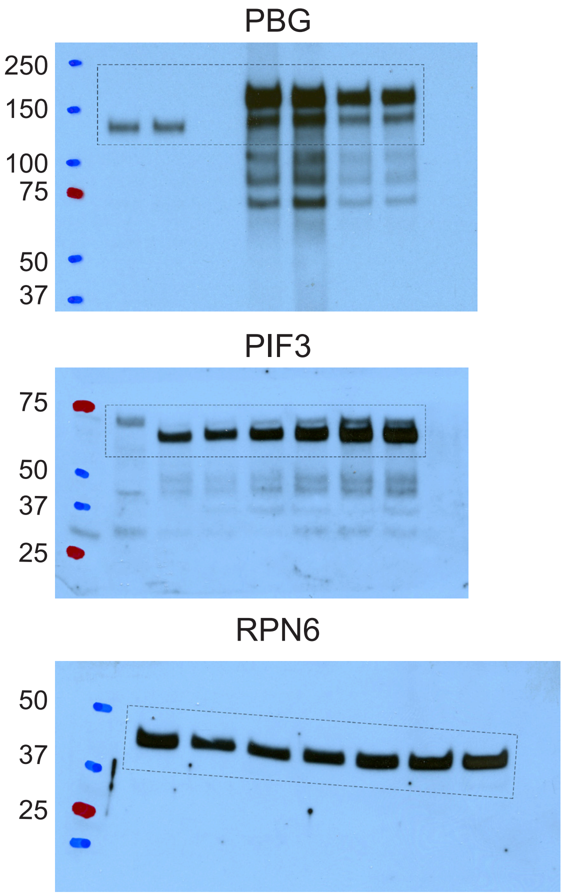


**Supplementary Figure 4. (continued)**

**Supplementary Table 1.** Primers used for making constructs.

| Name | Vector | Forward primer | Reverse primer |
|---|---|---|---|
| BD-HKRD | pGBKT7 | CACGGCCATATGGGCCAAGATACGGATAAGTTC | GGCGGATCCATATGGCATCATCAGCATC |
| AD-HKRD | pGADT7AD | CACGGCCATATGGGCCAAGATACGGATAAGTTC | GGCGGATCCATATGGCATCATCAGCATC |
| BD-HKRD18 | pGBKT7 | CACGGCCATATGGGCCAAGATACGGATAAGTTC | GGCGGATCCATATGGCATCATCAGCATC |
| AD-HKRD18 | pGADT7AD | CACGGCCATATGGGCCAAGATACGGATAAGTTC | GGCGGATCCATATGGCATCATCAGCATC |
| BD-HKRD-D | pGBKT7 | CATATGGCCATGGAGGCCGCTGGCCAAGATACGGATAAGTTC | CGGCCGCTGCAGGTCGACGTCATGGTGCATACCGGATTATACTC |
| AD-HKRD-D | pGADT7AD | TATGGCCATGGAGGCCAGTGCTGGCCAAGATACGGATAAGTTC | TCTGCAGCTCGAGCTCGATGTCATGGTGCATACCGGATTATACTC |
| BD-HKRD-CA1 | pGBKT7 | CATATGGCCATGGAGGCCGCTCAGAAGCAGTTACTTGAAACAAG | CGGCCGCTGCAGGTCGACGTCAATATGGCATCATCAGCATCATG |
| AD-HKRD-CA1 | pGADT7AD | TATGGCCATGGAGGCCAGTGCTCAGAAGCAGTTACTTGAAACAAG | TCTGCAGCTCGAGCTCGATGTCAATATGGCATCATCAGCATCATG |
| BD-HKRD-CA2 | pGBKT7 | CATATGGCCATGGAGGCCGCTGTGCTAAAGAGGGAAGAGTTTTTC | CGGCCGCTGCAGGTCGACGTCAATATGGCATCATCAGCATCATG |
| AD-HKRD-CA2 | pGADT7AD | TATGGCCATGGAGGCCAGTGCTGTGCTAAAGAGGGAAGAGTTTTTC | TCTGCAGCTCGAGCTCGATGTCAATATGGCATCATCAGCATCATG |
| BD-HKRD-CA3 | pGBKT7 | CATATGGCCATGGAGGCCGCTCAGATAAGGATTCAACAGCTCC | CGGCCGCTGCAGGTCGACGTCAATATGGCATCATCAGCATCATG |
| AD-HKRD-CA3 | pGADT7AD | TATGGCCATGGAGGCCAGTGCTCAGATAAGGATTCAACAGCTCC | TCTGCAGCTCGAGCTCGATGTCAATATGGCATCATCAGCATCATG |
| Myc-PHYB | pGBKT7 | CATATGGCCATGGAGGCCGCTATGGTTTCCGGAGTCGGG | CGGCCGCTGCAGGTCGACGTCAATATGGCATCATCAGCATCATG |
| HA-PHYB | pGADT7AD | TATGGCCATGGAGGCCAGTGCTATGGTTTCCGGAGTCGGG | TCTGCAGCTCGAGCTCGATGTCAATATGGCATCATCAGCATCATG |
| Myc-PHYB18 | pGBKT7 | CATATGGCCATGGAGGCCGCTATGGTTTCCGGAGTCGGG | CGGCCGCTGCAGGTCGACGTCAATATGGCATCATCAGCATCATG |
| HA-PHYB18 | pGADT7AD | TATGGCCATGGAGGCCAGTGCTATGGTTTCCGGAGTCGGG | TCTGCAGCTCGAGCTCGATGTCAATATGGCATCATCAGCATCATG |
| Myc-BC | pGBKT7 | CATATGGCCATGGAGGCCGCTAACTCTAAAGTTGTGGATGGTG | CGGCCGCTGCAGGTCGACGTCAATATGGCATCATCAGCATCATG |
| HA-BC | pGADT7AD | TATGGCCATGGAGGCCAGTGCTAACTCTAAAGTTGTGGATGGTG | TCTGCAGCTCGAGCTCGATGTCAATATGGCATCATCAGCATCATG |
| Myc-BC18 | pGBKT7 | CATATGGCCATGGAGGCCGCTAACTCTAAAGTTGTGGATGGTG | CGGCCGCTGCAGGTCGACGTCAATATGGCATCATCAGCATCATG |
| HA-BC18 | pGADT7AD | TATGGCCATGGAGGCCAGTGCTAACTCTAAAGTTGTGGATGGTG | TCTGCAGCTCGAGCTCGATGTCAATATGGCATCATCAGCATCATG |
| PHYB-HA | pCMX-PL2-CterHA | GGCGGTACCATGGTTTCCGGAGTCGGG | GCAGCCCGGGATATGGCATCATCAGCATCATG |
| PHYB18-HA | pCMX-PL2-CterHA | GGCGGTACCATGGTTTCCGGAGTCGGG | GCAGCCCGGGATATGGCATCATCAGCATCATG |
| BC-HA | pCMX-PL2-CterHA | AGATATCAGGATCGGTACATGAACTCTAAAGTTGTGGATGG | GATCCCCCGGGCTGCAGGGCATATGGCATCATCAGCATCATG |
| BC18-HA | pCMX-PL2-CterHA | AGATATCAGGATCGGTACATGAACTCTAAAGTTGTGGATGG | GATCCCCCGGGCTGCAGGGCATATGGCATCATCAGCATCATG |
| AC-Myc | pCMX-PL2-CterMyc | AGATATCAGGATCGGTACATGACAAGGAGTTTACCTTGGAAGGAC | GATCCCCCGGGCTGCAGGGCCTTGTTTGCTGCAGCGAGTTC |
| CC-Myc | pCMX-PL2-CterMyc | AGATATCAGGATCGGTACATGTGGAAAAGTGTGCCCTGGG | GATCCCCCGGGCTGCAGGGCAATCAAGGGAAATTCTGTGAGG |
| DC-Myc | pCMX-PL2-CterMyc | AGATATCAGGATCGGTACATGAGCCGATGTCAGCCATGG | GATCCCCCGGGCTGCAGGGCTGAAGAGGGCATCATCATCATTAG |
| EC-Myc | pCMX-PL2-CterMyc | AGATATCAGGATCGGTACATGAGCAGGAGCTTGCCGTGG | GATCCCCCGGGCTGCAGGGCCTTTATGCTTGAACTACCCTCTG |
| PBY18 | pCHF3-YFP | GGCGGTACCATGGTTTCCGGAGTCGGG | GGCGGTACCATATGGCATCATCAGCATCATG |
| PBY18N | pCHF3-YFP-NLS | GGCGGTACCATGGTTTCCGGAGTCGGG | GGCGGTACCATATGGCATCATCAGCATCATG |
| BCY18 | pCHF3-YFP | GGCGGTACCATGAGCCGGAGTCAGCCATGGG | GGCGGTACCATATGGCATCATCAGCATCATG |
| BCY18N | pCHF3-YFP-NLS | GGCGGTACCATGAGCCGGAGTCAGCCATGGG | GGCGGTACCATATGGCATCATCAGCATCATG |

**Supplementary Table 2.** qRT-PCR primers for the selected photosynthetic and auxin-related genes.

| Accession | Gene name | Forward primer | Reverse primer |
|---|---|---|---|
| AT1G69960 | *PP2A* | TATCGGATGACGATTCTTCGTGCAG | GCTTGGTCGACTATCGAATGAGAG |
| AT1G29920 | *CAB2* | TGAGCCAAGTTCTATCTGTTTG | TCTACCATCCACCACAAACAC |
| AT2G34430 | *LHB1B1* | TCCTGCTTTGACCGGAAAGG | GGCTTTGCGCATGGTGATTC |
| AT2G05070 | *LHCB2.2* | TTATGGCCACATCAGCTATCC | CCAAGACGCCCACCTTTTG |
| AT3G54890 | *LHCA1* | TCTGCTCCTGGTGACTTTGG | CCAGGAACAGCGAGCATAG |
| AT5G18060 | *SAUR23* | ATTCAAACTTTCAGACAAAAGAAATGG | ACAAGGAAACAACTCTATCTCTAACT |
| AT5G18010 | *SAUR19* | CTTCAAGAGCTTCATAATAATTCAAACTT | GAAGGAAAAAATGTTGGATCATCTT |
| AT3G15540 | *IAA19* | ATCGGTGTGGCCTTGAAAG | AACATCCCCCAAGGTACATC |
| AT1G52830 | *IAA6* | GTGCCATACATGAGGAAGATTG | CCACTCCTATGCCAAGACAG |
