# Supplementary material for: Mechanism of early light signaling by the carboxy-terminal output module of Arabidopsis phytochrome B
Source: Nat Commun. 2017 Dec 4;8:1905. doi: 10.1038/s41467-017-02062-6 (PMC5712524; doi:10.1038/s41467-017-02062-6)
Supplement: Supplementary file 3 — Description of Additional Supplementary Files [file 41467_2017_2062_MOESM3_ESM.pdf]

Description of Supplementary Files

File Name: Supplementary Data 1
Description: BCY- and PIF-coregulated gene list.

File Name: Supplementary Data 2
Description: GO enrichment analysis of the BCY- and PIF-coregulated genes.
